# Supplementary material for: Sugary Liquids in the Baby Bottle: Risk for Child Undernutrition and Severe Tooth Decay in Rural El Salvador
Source: Int J Environ Res Public Health. 2020 Dec 31;18(1):260. doi: 10.3390/ijerph18010260 (PMC7795345; doi:10.3390/ijerph18010260)
Supplement: Supplementary file 1 [file ijerph-18-00260-s001.pdf]

File S1: ASAPROSAR Mother Interview Form (2010)

Entrevista de la Madre

Unique ID:
Fecha (m/d/a):
Nombre de la madre:

- ¿Si la madre no dió la entrevista, lo quien dió? (nombre y relación)

Promotora:
Pueblo:

1. Su edad (#)
2. Su nivel de educación (#, 0-18)
3. ¿Cuántos hijos tiene? (#)
4. ¿Cuántos son menores de 7 años? (#)

Nombres de hijos/as menores de 7 años?	Edades

5. ¿Cuántas personas viven en su casa?
6. ¿Su casa tiene luz eléctrica?
    a. Sí
    b. No
7. ¿Su casa tiene agua potable?
    a. Sí
    b. No
8. ¿Cocina con leña, gas, o los dos?
    a. Leña
    b. Gas
    c. Leña y gas

[Instrucciones por la promotora] Ahora tengo algunas preguntas acerca de su salud:

9. ¿Cuándo va usted al doctor?
    a. Solo cuando está enferma
    b. Para chequeos
    c. Los dos
    d. En verdad, nunca se va al doctor
10. ¿Cuándo va usted al dentista?
    a. Solo cuando está mal con los dientes/muelas
    b. Para chequeos
    c. Los dos
    d. En verdad, nunca se va al dentista
11. ¿Cada cuanta toma usted leche?
    a. Nunca
    b. Cada 2-4 semanas
    c. Cada semana
    d. 2-3 veces a la semana
    e. 1 vez al día
    f. 2-3 veces al día
12. ¿Cada cuanta toma usted gaseosas?
    a. Nunca
    b. Cada 2-4 semanas
    c. Cada semana
    d. 2-3 veces a la semana
    e. 1 vez al día
    f. 2-3 veces al día
13. ¿Cuántos dientes o muelas se le han caído o le han extraído en su vida? (#)
14. Ahora en su casa, ¿tiene usted su propio cepillo de dientes?
    a. Si
    b. No
15. ¿Hace cuánto tiempo fue usted al dentista? (meses, #)
    a. Nunca se fue, = 00
16. ¿Porqué fue al dentista la última vez?
    a. Dolor del diente/muela
    b. Sangramiento de las encías
    c. Dientes/muelas podridos
    d. Relleno del diente/muela
    e. Extracción del diente/muela
    f. Un chequeo/control
    g. Porque fui con mi hijo/a
17. ¿En los últimos 3 meses, ha tenido usted algún problema con los dientes, las muelas, las encías, o la boca? (marque todos los que apliquen)

a. Dolor o sensibilidad
b. Dientes/muelas podridos/sueltos
c. Sangramiento de las encías
d. Inflamación en la boca
e. Otras: _______

[Instrucciones por la promotora] (18-20, No lée las respuestas):

18. ¿Qué piensa ud.—de que vienen las caries en los dientes de leche?
    a. No sé
    b. Dulces
    c. Chicles
    d. Jugo/soda
    e. No cepillarse
    f. Pacha
    g. Otras: _______
19. ¿Piensa ud. que las caries causan problemas para los niños?
    a. Sí
    b. No
    c. No sé
20. ¿Cómo les afectan a sus hijos?
    a. No sé
    b. Dolor
    c. No pueden comer
    d. No pueden dormir
    e. Dientes/muelas podridos
    f. Daña su salud

Información del Niño

[Instrucciones por la promotora] Ahora vamos a hacer preguntas acerca del hijo/a que se está examinando:

ID del Niño:

21. Nombre del niño:
22. Fecha de nacimiento (m/d/a):
23. Sexo
    a. M

    b. F
24. ¿Recibió controles prenatales cuando estaba embarazada con este bebé?
    a. Si
    b. No
25. ¿Cuántos controles? (#)
26. Las vacunas del niño/a están completeas?
27. ¿Le dio pecho a su bebé?
    a. Si
    b. No
    c. No sé
28. Si le dio pecho, ¿hasta qué edad en meses? (#)
    a. ¿Sigue tomando pecho?
        i. Sí
        ii. No
29. Su hijo/a tomó pacha?
    a. Sí
    b. No (→ 33)
30. Si tomó pacha, ¿hasta qué edad? (en meses, #)
    a. ¿Sigue tomando pacha?
31. ¿Cada cuanto dormía con la pacha en la boca?
    a. Nunca
    b. De repente
    c. Frecuentemente
    d. Casi siempre
32. ¿Qué tomaba en la pacha (marque todos los que apliquen)?
    a. Agua
    b. Leche
    c. Formula
    d. Limonada
    e. Otro jugo natural
    f. Jugo artificial/frescos
    g. Café
    h. Gaseosa/soda
    i. Agua azucarada
    j. Atol
    k. Incaparina
    l. Otras: ______

¿Cada cuánto consume su hijo las siguientes cosas?

33. Leche
    a. Nunca
    b. Cada 2-4 semanas
    c. Cada semana
    d. 2-3 veces a la semana
    e. 1 vez al día
    f. 2-3 veces al día
34. Gaseosas
    a. Nunca
    b. Cada 2-4 semanas
    c. Cada semana
    d. 2-3 veces a la semana
    e. 1 vez al día
    f. 2-3 veces al día
35. Dulces/caramelos
    a. Nunca
    b. Cada 2-4 semanas
    c. Cada semana
    d. 2-3 veces a la semana
    e. 1 vez al día
    f. 2-3 veces al día

[Instrucciones por la promotora] (36-37, No lée las respuestas):

36. Cuando su hijo/a chiquito/a está llorando durante el día o la noche, qué hace usted para calmarlo/a?
    a. Contemplarle
    b. Pegarle
    c. Darle medicina
    d. Darle un dulce
    e. Darle comida
    f. Darle el pecho
    g. Darle la pacha
    h. Otras: ________
37. Qué hace usted para cuidar los dientes de su hijo/a?
    a. Cepillarse
    b. No darle dulces
    c. Nada
    d. Otras: __________

38. Ahora en su casa, tiene su hijo/a su propio cepillo de dientes?
    a. Sí
    b. No
39. Ahora en su casa, tiene su hijo/a pasta dental?
    a. Sí
    b. No
40. Usted le ayuda a su hijo/a a cepillarse?
    a. Nunca
    b. De repente
    c. Frecuentemente
    d. Casi siempre
41. Su hijo/a ha ido al dentista?
    a. Sí
    b. No (→ 44)
42. Si ha ido al dentista, ¿cuántas veces? (#)
43. Si ha ido al dentista, ¿porqué fue?
    a. Para chequeos
    b. Caries
    c. Dolor
    d. Otras: _______
44. Cada cuanto dice su hijo/a que le duele la boca/muelas/dientes?
    a. Nunca
    b. De vez en cuando
    c. Frecuentemente
    d. Casi siempre
45. ¿Cada cuanto tiene problemas en comer por dolor en la boca?
    a. Nunca
    b. De vez en cuando
    c. Frecuentemente
    d. Casi siempre
46. ¿Cada cuanto llora en la noche por dolor en la boca?
    a. Nunca
    b. De vez en cuando
    c. Frecuentemente
    d. Casi siempre
47. En su opinión, cómo están los dientes de su hijo/a?
    a. Excelentes
    b. Más o menos buenos
    c. Malos
48. Cómo está su hijo/a de salud?

   a. Muy sano
   b. Más o menos
   c. Muy enfermo

49. Si su hijo/a ha ido al dentista en el último año, ¿qué le hizo?
